# Fitness Trade-Offs in the Evolution of Dihydrofolate Reductase and Drug Resistance in *Plasmodium falciparum*

**DOI:** 10.1371/journal.pone.0019636

**Published:** 2011-05-23

**Authors:** Marna S. Costanzo, Kyle M. Brown, Daniel L. Hartl

**Affiliations:** Department of Organismic and Evolutionary Biology, Harvard University, Cambridge, Massachusetts, United States of America; Menzies School of Health Research, Australia

## Abstract

**Background:**

Patterns of emerging drug resistance reflect the underlying adaptive landscapes for specific drugs. In *Plasmodium falciparum*, the parasite that causes the most serious form of malaria, antifolate drugs inhibit the function of essential enzymes in the folate pathway. However, a handful of mutations in the gene coding for one such enzyme, dihydrofolate reductase, confer drug resistance. Understanding how evolution proceeds from drug susceptibility to drug resistance is critical if new antifolate treatments are to have sustained usefulness.

**Methodology/Principal Findings:**

We use a transgenic yeast expression system to build on previous studies that described the adaptive landscape for the antifolate drug pyrimethamine, and we describe the most likely evolutionary trajectories for the evolution of drug resistance to the antifolate chlorcycloguanil. We find that the adaptive landscape for chlorcycloguanil is multi-peaked, not all highly resistant alleles are equally accessible by evolution, and there are both commonalities and differences in adaptive landscapes for chlorcycloguanil and pyrimethamine.

**Conclusions/Significance:**

Our findings suggest that cross-resistance between drugs targeting the same enzyme reflect the fitness landscapes associated with each particular drug and the position of the genotype on both landscapes. The possible public health implications of these findings are discussed.

## Introduction

*Plasmodium falciparum*, the causative agent of the most virulent form of malaria, has demonstrated an astounding ability to rapidly evolve resistance to many different classes of drugs. Drug resistance in malaria is thus a zealously investigated topic, covering everything from developing methods to detect emerging drug resistance to designing drugs that may prolong usefulness in the face of rapidly evolving resistance. Discovering the adaptive landscape of drug resistance provides another way to gain an understanding of how antimalarial drug resistance evolves and how the course of evolution is shaped by molecular forces.

One class of antimalarials, the antifolates, works by competitively inhibiting enzymatic action in the folate pathway. Within the folate pathway the enzymes dihydrofolate reductase (DHFR) and dihydropteroate synthase (DHPS) are most often targeted for inhibition. DHFR performs the essential task of reducing dihydrofolate to tetrahydrofolate [1], which is a cofactor in the synthesis of purines, pyrimidines, and amino acids [2]–[3]. DHPS is also a key component in the biosynthesis of folate, producing the product 7,8-dihydropteroate which immediately preceeds folate in the pathway [4]. Antifolates targeting DHFR are often combined with sulfa-drugs aimed at DHPS and are used in combination therapies such as sulfadoxine-pyrimethamine (SP) and trimethoprim-sulfamethoxazole due to the synergistic properties of such combinations. The folate pathway is an excellent drug target due to the indispensable nature of the products of the pathway and due to the presence of fixed differences in the amino acid sequence between parasite and host that allow targeted inhibition of species-specific variants of DHFR. In the case of malaria, the species-specific differences mean that administering an antifolate drug can kill the parasite without excessive harmful side-effects to the human host.

For decades the great efficacy, stability, and cost-effectiveness of the antifolate pyrimethamine had made it the first-line of defense in many developing countries plagued by malaria [5]. Regrettably, resistance to pyrimethamine and other antifolates such as proguanil and chlorproguanil has become widespread. While sulfadoxine-pyrimethamine is still being used as Intermittent Preventative Treatment (IPT) in pregnant women, although even in IPT resistance to sulfadoxine pyrimethamine has become a concern [6]. SP has thus been largely been replaced by artemisinin combination therapies (ACT) in clinical settings. Antifolate resistance is based on a handful of point mutations in *pfdhfr*, the *P. falciparum* gene coding for dihydrofolate reductase (DHFR) that may be compounded by concurrent resistance in DHPS (see [6] for review of antifolate resistance).

Mutations in DHFR, which may occur in combination with mutations in DHPS, are now widely observed in the field, and pyrimethamine is fast losing efficacy as a therapeutic agent due to this rapidly evolving drug resistance [7]. The antifolate chlorproguanil is a candidate for replacing pyrimethamine in combination therapies. In the human body chlorproguanil is metabolized into the active compound chlorcycloguanil, which functions as a competitive inhibitor of DHFR in a manner similar to that of pyrimethamine. Chlorcycloguanil is a biguanide, like cycloguanil, but possesses an additional chlorine group on the benzene ring. Chlorproguanil was, until recently, a component in a promising new drug combination comprising chlorproguanil-dapsone (LapDap®, GlaxoSmithKline), which was withdrawn due to side-effects associated with dapsone [8].

Chlorcycloguanil-based therapies have potent antimalarial activity, a short half-life, and have been shown to exert decreased selective pressure for drug-resistance when compared to pyrimethamine [9]–[10], which makes them an attractive alternative to pyrimethamine in new combination therapies [11]. However, cross-resistance among antifolates is still of great concern and, in the case of chlorcycloguanil and pyrimethamine, the existence of apparent cross-resistance was described several decades ago [12] and has also been examined more recently [13].

The molecular basis for antifolate resistance is well understood. Specific amino acid changes at codons 16 (alanine to valine, A16V), 51 (asparagine to isoleucine, N51I), 59 (cysteine to arginine, C59R), 108 (serine to asparagine or threonine, S108N/T), and 164 (isoleucine to leucine, I164L) are known to greatly increase antifolate resistance [14]–[16]. Certain of these mutations are associated with resistance to a particular antifolate. Thus A16V in combination with S108T is associated with resistance to cycloguanil (the active metabolite of proguanil) but not to pyrimethamine resistance, while cross-resistance to both cycloguanil and pyrimethamine is associated with alleles that include both S108N and I164L[17]. The triple DHFR mutant S108N-N51I-C59R is associated with sulfadoxine-pyrimethamine treatment failure. Patients that are infected with parasites exhibiting the DHFR triple mutant in combination with a DHPS double mutant at positions 437 (alanine to glycine, A437G) and 540 (lysine to glutamic acid, K540E) have an even higher risk for early treatment failure than with the triple DHFR mutant alone [18].

Moreover, the laboratory findings that show genotypes containing one or more of these mutations to confer antifolate resistance correlate with the molecular markers associated with resistance to one or multiple drugs in the field. Thus, *in vitro* studies show that a triple mutant N51I/C59R/S108N increases resistance to pyrimethamine 80 fold and resistance to chlorcycloguanil 40 fold [15]. In clinical settings, *P. falciparum* strains carrying these same mutations (N51I/C59R/S108N) are associated with high rates of treatment failure using sulfadoxine-pyrimethamine but remain treatable by chlorproguanil-dapsone [19]. The addition of a mutation of I164L to the triple mutant leads to chlorcycloguanil resistance in addition to pyrimethamine resistance [20].

Although much is known about the genes and mutations leading to chlorcycloguanil resistance, genetics in malaria parasites remains technically challenging thus limiting the scope of research possible in live parasites. The development of heterologous expression systems for *pfdhfr* in yeast [21] and bacteria [22] have allowed for more extensive research into antifolate resistance. Using transgenic methods, novel alleles conferring drug resistance to pyrimethamine and chlorcycloguanil have been identified by targeted mutagenesis on *pfdhfr* [23]–[24], and novel alleles leading to multi-drug resistance against pyrimethamine and chlorcycloguanil have been described [25]. These systems also provide a tractable way to examine evolution, and differences in selection pressures on *pfdhfr* have been described between the antifolates pyrimethamine and WR99210 [26].

More recent studies have sought to understand the evolutionary trajectories that may lead to antifolate drug resistance in this system, focusing on resistance to pyrimethamine. The evolution of pyrimethamine resistance has been modeled at the molecular level in yeast [27] and in a comparable system in bacteria [28]. These studies found that pathways leading to maximum resistance are limited, showed that some initial loss in fitness is restored by compensatory mutations in later steps of the evolutionary process, and implied that the withdrawal of pyrimethamine use is not likely to restore its efficacy as a therapeutic compound.

The aim of the current study is to use the relative resistance of mutant alleles of the *pfdhfr* gene to chlorcycloguanil in a yeast system in order to explore the evolutionary landscape underlying chlorcycloguanil resistance. We assayed all possible combinations of 6 mutations at 5 amino acid residues (codons 16, 51, 59, 108, and 164) and explored the evolutionary pathways accessible from the drug-susceptible wildtype allele to strongly resistance conferring mutant alleles. We also consider the implications of chlorcycloguanil resistance in the light of known patterns of pyrimethamine resistance caused by mutations in the same codons.

## Results

### Growth rate

**Figure 1:**
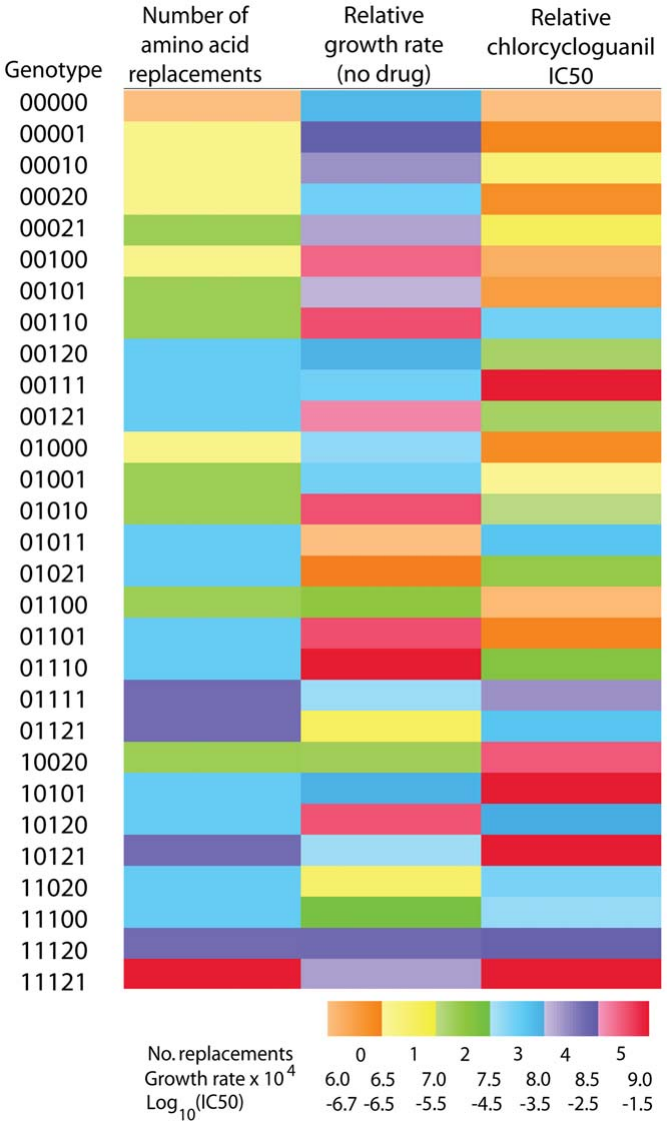
This heatmap indicates the relationship between the number of amino acid replacements, growth rate in the absence of drug (at an OD of 600 nm) and drug resistance (IC50). Genotypes are indicated on the left hand side. No clear correlation is seen between the number of mutations and growth rate or resistance.

Of the 48 alleles constructed, 29 were viable and able to grow in the absence of metabolic supplement (Figure 1 and Table S6 in File S1). Previous experiments have indicated variations in growth rate among mutant alleles of *pfdhfr* as expressed in a yeast system [25] [27]. Consistent with those results, growth rate analysis on our data shows differences in growth rate among the mutant alleles in the absence of drugs, with mutants growing both better and worse than the wildtype strain.

There is no discernable relation between the number of mutations in an allele and its viability (Figure 1 and Table S2 in File S1.). The A16V change is the single most detrimental mutation, with 16 non-viable alleles out of a possible 24 alleles where A16V was present. Six of the eight viable alleles with an A16V mutation also carry S108T.

### IC50

Figure 1 shows the variation among IC50 values obtained for the different alleles and how the IC50s relate to growth rate and number of mutations. Numerical IC50 values are presented in Table S7 in File S1. Four of the alleles proved to be highly resistant to chlorcycloguanil. These were the quintuple mutant 11121 (A16V/N51I/C59R/S108T/I164L), the quadruple mutant 10121 (A16V/C59R/S108T/I164L), and the two triple mutants 00111 (C59R/S108N/I164L) and 10101 (A16V/C59R/I164L). Under our experimental conditions, the high IC50 values for chlorcycloguanil for these alleles were not significantly different. All of these alleles do, however, show a significantly higher IC50 than the allele ranked fifth, which is the double mutant 10020 (A16V/S108T).

The mutation A16V, which exhibits a non-viable phenotype when present on a wildtype background, is associated with drug resistance on a background containing S108T, and in this background A16V increases chlorcycloguanil resistance 10,000 fold.

In order to rank the four most highly resistant alleles for purposes of the simulations, we estimated the IC25 and IC2 values using the non-linear regression values obtained from the same analysis used for estimating the IC50s (see Table S3 in File S1). The IC2 was also estimated independently using the linear regression values for the slope between the two points bracketing a 2% decrease in growth rate from the no-drug growth [24], as these values had been explicitly measured for the relevant strains (see Table S4 in File S1). Estimation of IC25 and IC2 values require a smaller decrease in growth rate (25% and 2% respectively) making it a useful tool in ranking strains that are so resistant that larger decreases in growth rate is difficult to attain for the purpose running simulations that require ranking. However, caution should be used in applying these rankings for any other purpose as the small changes are necessarily confounded by error rates that are relatively large.

### Evolutionary trajectories

**Figure 2:**
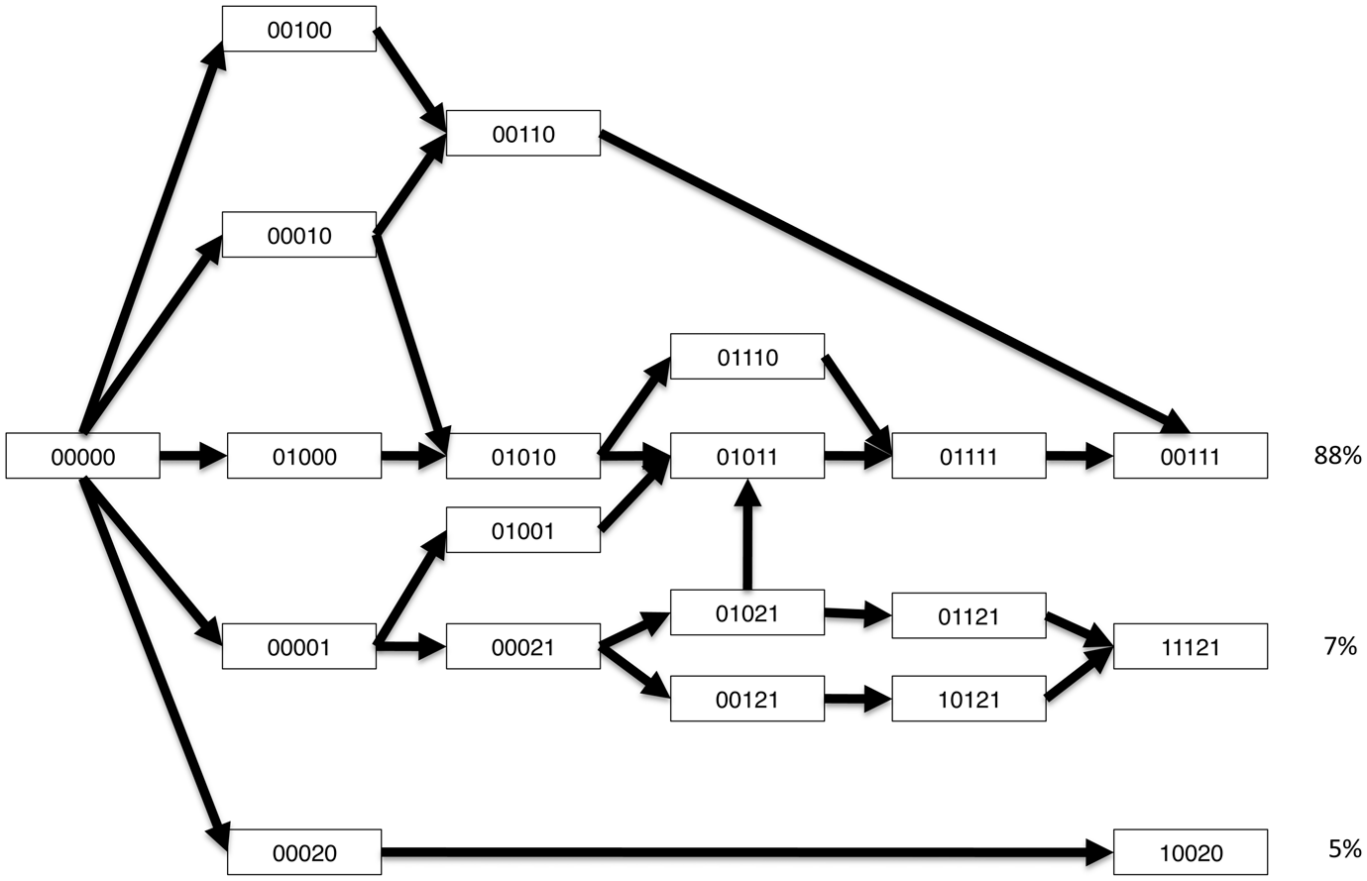
The top ten most often realized evolutionary trajectories. The trajectories move from the wildtype allele (00000) to one of three endpoints (00111, 10020, or 111121) in steps of one mutation at a time. The percentage of trajectories that lead to each endpoint is indicated. The genotype 00111 is the endpoint of the majority of trajectories.

The 83 evolutionary trajectories that were accessible during the simulations are summarized in Table S5 in File S1. Simulations were run on 750 landscapes and with 100,000 rounds of evolution on each. The top five most frequently accessed trajectories account for approximately 80% of the probability for pathway realization, while the top ten most often accessed trajectories make up 92% of that probability. The landscape is multi-peaked and three endpoints are accessible: the double mutant 10020 (A16V/S108T), the triple mutant 00111 (C59R/S108N/I164L), and the quintuple mutant 11121 (A16V/N51I/C59R/S108T/I164L) (Figure 2). Pathways leading to the double mutant 10020, which ranks fifth in IC50 overall, accounted for 7% of all realizations; those leading to the triple mutant 00111 account for 88% of realizations; and those leading to the quintuple mutant 11121 account for 5% of all accessible pathways. As noted, the triple mutant 00111 and the quintuple mutant 11121 have IC50 values that are very high and indistinguishable under our experimental conditions, and hence the simulated evolutionary trajectories terminate in high-IC50 alleles 93% of the time.

The single most often accessed trajectory (00000-00010-00110-00111) is observed on all 750 landscapes, and accounts for 20% of all realized trajectories. The most frequently followed trajectory to the double mutant 10020 (A16V/S108T) is 00000-00020-10020, which is followed in about 6% of simulations. The most frequently followed trajectory to the quintuple mutant 11121 (A16V/N51I/C59R/S108T/I164L) is 00001-00021-01021-01121-11121 and accounts for approximately 2% of realizations.

## Discussion

There is a long-standing and continuing interest in resistance to antifolates and the level of cross-resistance observed between different antifolates [29]. The relationship between resistance to pyrimethamine and resistance to cycloguanil [20] [15], the active metabolite of proguanil, has been investigated, and to a lesser extent that between pyrimethamine and chlorcycloguanil, the active metabolite of chlorproguanil [13] [9]. We use previously published analysis of the landscape of pyrimethamine resistance [27] along with new data on chlorcycloguanil from the current study to analyze the underlying evolutionary landscape of drug resistance for these compounds. Our analysis shows that, while some alleles show increased resistance to chlorcycloguanil as well as pyrimethamine, the adaptive landscapes underlying the evolution of drug resistance have unique features associated with each antifolate specifically and are not merely a function of changes at the sequence level of the enzyme being challenged.

Common to pathways of pyrimethamine and chlorcycloguanil resistance are the triple mutant 00111 (C59R/S108N/I164L) and the quadruple mutant 01111 (N51I/C59R/S108N/I164L), both of which increase resistance significantly in the presence of either compound. In contrast, the beneficial effect of the double mutant 10020 (A16V/S108T) differs greatly in magnitude between pyrimethamine (two fold increase) and chlorcycloguanil (10,000 fold increase). Evolutionary analysis showed that this double mutant constitutes a fitness peak on the chlorcycloguanil landscape, and this submaximal fitness may be reached in only two mutational steps. In contrast, 10020 is an allele on the pyrimethamine landscape that is so rarely traversed that it does not show up in simulations. This example serves to illustrate the importance of understanding not just the genetic basis of resistance, but also the fitness landscape in the presence of a particular selective agent, in this case the specific antifolate drug.

Looking at practical applications of doing such simulations for more than one drug, one could imagine using genotypic information of the alleles currently present in parasites infecting a patient (or at alleles present the population level) and establishing if whether those alleles are fitness peaks on second line drugs that are being considered. In other words, in a scenario where chlorcycloguanil has been the major selective force and the presence of the 10020 allele has led to strong drug resistance, pyrimethamine would still be effective. However, if chlorcycloguanil resistance is conferred by amino acid substitutions of the 00111 type, the occurrence of a single mutation to 01111 would render both drugs ineffective. Of course, further systematic corroboration with clinical data is necessary before this scenario can safely determine care for human patients, but the possibility for the eventual fruitful application of *in vitro* resistance assays and *in silico* landscape simulation certainly exists.

Interestingly, all four of the alleles with the highest IC50s include the I164L mutation. The addition of I164L on certain backgrounds is associated with as much as a 1000-fold increase in resistance to antifolates, including chlorcycloguanil [25]. In the field, I164L is associated with the majority of treatment failures that occur when using a chlorcycloguanil combination therapy [30] [31] [18]. I164L is known to be a late arising mutation in the emergence of drug resistance in clinical settings, where the more widely used pyrimethamine is likely to have played a much greater role in shaping the evolution of drug resistance than the less ubiquitously used chlorcycloguanil. Looking at I164L as a component of mutational trajectories, it occurs as the first step in only 2% of trajectories on the pyrimethamine landscape [27] which is congruent with the relative scarcity of this mutation in natural settings as a responcse to pyrimethamine drug pressure. However, 14% of trajectories (leading to both the 00111 and 11121 peaks) on the chlorcycloguanil landscape have I164L as a first step. This finding underscores the importance of the I164L mutation in leading to high-level resistance to chlorcycloguanil specifically.

Our findings further demonstrate that, based on the chlorcycloguanil landscape in yeast, I164L is not necessarily a late arising mutation. This likely reflects a fundamental difference between the adaptive landscapes for pyrimethamine and chlorcycloguanil and underscores the importance of drug specific patterns of evolution. Simultaneously we recognize that I164L may not be as readily found as a single mutation in nature due to some additional fitness deficit that is not related to selection by pyrimethamine use but which we cannot determine in this context. It has already been observed that early mutations may entail some loss of fitness under a regime where no drug pressure is present but fitness is later restored by compensatory mutations [28]. Trajectories containing I164L as an initial step seem to follow such a pattern. An important observation is gained from the startling presence of I164L among the initial mutational steps found on among many trajectories. That is, the probability of evolutionary trajectories and the order of the steps that make up those trajectories can be heavily influenced by differences in the underlying landscape based on the drug treatment used.

The second most often accessed endpoint is the quintuple mutant 11121 (A16V/N51I/C59R/S108T/I164L), an allele not generally observed in clinical settings. Conceivably this may simply be due to a smaller sample size for alleles associated with chlorcycloguanil resistance. Chlorcycloguanil is not and has not been used as widely as pyrimethamine, and there is less data available on resistance mutations in the field apart from clinical trials. However, there are also intrinsic explanations for this observation. As two more mutations are required to reach this allele, it may be expected to occur more infrequently than a triple mutant given that mutations at all positions are equally likely. Alternatively, the relative dearth of pathway realizations leading to the quintuple mutant 11121 may be a reflection of the negative interaction between the mutations found in this allele. This allele contains the mutation S108T, which is much more rarely observed than S108N and usually occurs in combination with A16V [32]. It has already been noted that alleles containing A16V are often non-viable although some are rescued by the addition of S108T.

The order in which mutations occur may thus play a critical role in determining whether a particular path leading to 11121 is accessible by evolution, or whether a fitness valley makes this trajectory impassable. Accessing a path of increasing resistance to the triple mutant 00111 (C59R/S108N/I164L) does not have the same problem, as this allele contains mutations that ”mix” much better. Due to the effect of such epistatic interactions there may be a more limited number of viable alleles available as options when traversing the landscape from 00000 to 11121.

Interestingly, 10121 (A16V/C59R/S108T/I164L) and 10101 (A16V/C59R/I164L) exhibit high IC50s, indistinguishable from those of the triple mutant 00111 (C59R/S108N/I164L) and the quintuple mutant 11121 (A16V/N51I/C59R/S108T/I164L), but these alleles are not accessed as endpoints in the simulations. In the case of 10121 this is probably due to the adjacency in sequence space of the quintuple mutant 11121, which was assigned a higher rank based on our estimations. However, in all our simulations, 10101 (A16V/C59R/I164L) appears only once, and then as an intermediate step on a pathway leading to 11121 (00000-00100-00101-**10101**-10121-11121). Unlike the alleles on the more frequently traversed pathways to the fitness peak at 00111 (C59R/S108N/I164L), the allele 10101 is not one generally found in clinical settings. The trajectory analysis thus shows that the absence of this allele in nature is likely due to the unfavorable location of this point in sequence space, having both a low probability of realization and not occurring as the end point to a pathway, rather than due to higher susceptibility to drug treatment.

Cycloguanil-resistant/pyrimethamine-sensitive parasites have emerged in clinical settings [16]. In our data, the double mutant 10020 (A16V/S108T) shows how that situation can arise. The 10020 allele is a fitness peak on a landscape of resistance to chlorcycloguanil. However, a strain that evolves to the 10020 double mutant in response to selection by chlorcycloguanil would be susceptible to pyrimethamine.

The results of the evolutionary trajectory analysis further complement studies showing incomplete cross-resistance between chlorcycloguanil and pyrimethamine. In an *in vitro* study of *P. falciparum* field isolates, cross-resistance existed between pyrimethamine and chlorcycloguanil and the triple mutant (S108N/C59R/N51I or 01110) increased resistance to the drugs 225-fold and 48-fold, respectively [13]. While *in vitro* the presence of this triple DHFR mutant at codons 108, 51, and 59 (combined with a double DHPS mutant) increased drug resistance to both antifolates, this combination of mutations was only associated with clinical treatment failure with sulfadoxine-pyrimethamine but not with chlorproguanil-dapsone [18]. The addition of the I164L mutation results in clinical failure of chlorproguanil-based therapies.

In 1968 a study of the parasite *P. berghei* in mice showed that there is some cross-resistance between parasites exposed to chlorcycloguanil and pyrimethamine, with exposure to chlorcycloguanil inducing broader resistance than pyrimethamine [33]. It was clear then that certain resistant strains may be countered by using a different drug targeting the same mutant enzyme, but that multiply resistant lines may also arise. Tracing the mutational trajectories of those two drugs now affords an evolutionary explanation for those results from many decades ago: The shape of the underlying adaptive landscapes of the drugs in question affects the genotypes (and thus phenotypes) that will emerge—even between drugs targeting the same enzyme in the same manner. Drug resistance is a major factor in determining the course of treatment for malaria and other infectious diseases. The ability to not only pinpoint mutations that lead to drug resistant phenotypes, but also to predict the route evolution is most likely to take once drug pressure is exerted, are invaluable assets in preserving the efficacy of chemotherapy.

Knowledge regarding the progression of evolutionary trajectories can be determined in an *in vitro* system in a relatively short period of time as compared to tracking trends in the field. Scientists and clinicians may then have advance notice of the mutational progression in trajectories that are likely to lead to the exhibition of high drug resistance allowing them to use their limited resources in a more focused way as they survey for the emergence of drug resistance. Transgenic systems such as the one used in this study cannot address all the complexities involved in the evolution of drug resistance in clinical settings. An adaptive landscape established in a yeast system may thus not directly apply to natural populations of *P. falciparum*. Rather it provides a tractable way of exploring the processes governing patterns and constraint of drug evolution in nature and serves to inform further studies in the field.

## Materials and Methods

### Construction of yeast strains

Yeast *dhfr* knockout strains may be rescued by replacing the endogenous yeast gene with the *P. falciparum* gene. This creates a yeast strain that is sensitive to antimalarial drugs targeting DHFR and thus allows the study of the impact of mutations on drug sensitivity in a tractable manner. Genetic manipulation and drug resistance assays in *P. falciparum* remains technically challenging and thus *in vitro* experimentation can be very valuable if we keep in mind that this heterologous system may not in all cases accurately reflect the *in vivo* system. The construction of the *Saccharomyces cerevisiae* TH5 (*MATa leu2-3,112 trp1 ura3-52 dfr1::URA3 tup1*) strains containing mutant *Plasmodium falciparum dhfr (pfdhfr)* alleles is described in detail elsewhere [27]. Briefly, all 48 combinations of six mutations at five amino acid coding sites (Table S6 in File S1) in the *dhfr* gene of *P. falciparum* were constructed on the same genetic background using site-directed mutagenesis (QuikChange Site-Directed Mutagenesis Kit, Stratagene, Cedar Creek TX). Mutations were confirmed by sequencing. We transformed the yeast with each of the 48 alleles on a shuttle vector using the EZ Yeast Transformation Kit (Zymo, Orange CA). The presence of the GR7 vector was selected for by growing transformed colonies on tryptophan dropout media (SC trp^−^). The GR7 shuttle vector and the TH5 strain of *S. cerevisiae* were generously provided by Carol Sibley and were specifically engineered for use in *in vitro* assays to study antifolate resistance in *Plasmodium* [21].

### Growth rate and drug resistance assays

Growth rates and drug resistance assays were conducted as described previously [27] with the following alterations: After growing overnight at 30°C in 5 mL liquid YPD, colonies were diluted to an OD of 0.1 at 600 nm and then inoculated into 200 µL YPD containing chlorcycloguanil at concentrations of 0 M, 10^−8^ M, 10^−7^ M, 10^−6^ M, 10^−5^ M, and 10^−4^ M each. Chlorcycloguanil was diluted in ethanol to a final concentration of 1% ethanol. Chlorcycloguanil was generously provided by Jacobus Pharmaceuticals (Princeton, NJ). Growth rate and resistance experiments were conducted on a Bioscreen C microbiological workstation (Thermo Labsystems). Growth rates were measured by spectrophotometry at 600 nm at 30°C in 15 minute increments over a period of 72 hr.

### IC50 and calculation of evolutionary trajectories

The half-maximal inhibitory concentration (IC50) for each strain was determined using non-linear regression analysis in the open-source statistical environment R [34] using the method described in [27]. We used previously established methodology to determine the evolutionary trajectories that are accessible for DHFR evolution to attain resistance to chlorcycloguanil [35] [36] [27].

Our evolutionary model assumes that the time between mutations arising is much longer than the time to loss or fixation of new mutations, which allows the fixation of at most one mutation during each interval of time. At each interval, all mutational neighbors that will result in an increase in fitness are considered, including reversion to a previously fixed mutation [36]. It is assumed that the probability of fixation for beneficial mutations greatly outweighs that of deleterious or even neutral mutations, such that deleterious or neutral mutational fixations may be safely ignored in the presence of a possible beneficial mutation (see [27]) for complete details of this model).

We simulated landscapes and trajectories in this manner as it allows the probability estimates to account for uncertainty in our resistance measurements. Many trajectories thus occur on landscapes that have low-probabilities, i.e. the landscape itself has a low likelihood of realization. We therefore only report a consensus of trajectories that occur on at least 85% of landscapes. Doing so allows us to identify the strongest trends which is appropriate as in an *in vitro* system where some intricacies of *in vivo* systems may not be discernable but strong and important effects may still be observed in well planned experiments.

Using the IC50 values for the different mutant alleles, we ran computer simulations to determine the evolutionary trajectories most often accessed. Each initial condition consisted of a fitness landscape constructed by choosing a set of IC50 values, one for each genotype, by sampling one value at random from a normal distribution with the same mean and variance of IC50 as estimated for each genotype. For each of 750 such fitness landscape so constructed, 10^5^ independent computer simulations yielding a complete mutation-selection trajectory were carried out.

## Supporting Information

File S1 This chart shows the growth rates of all viable alleles in the absence of drug. The growth rates were determined by measuring cell density at OD600.
(DOCX)
