# Supplementary material for: Fitness Trade-Offs in the Evolution of Dihydrofolate Reductase and Drug Resistance in Plasmodium falciparum
Source: PLoS One. 2011 May 23;6(5):e19636. doi: 10.1371/journal.pone.0019636 (PMC3100297; doi:10.1371/journal.pone.0019636)
Supplement: File S1 — This chart shows the growth rates of all viable alleles in the absence of drug. The growth rates were determined by measuring cell density at OD600. (DOCX) [file pone.0019636.s001.docx]

Supporting material

**Table S.1**. The number of mutations in an allele is not strongly correlated with the viability of that allele (Pearson corr = 0.19).

**Mutations Viable Alleles Non-viable Alleles Total**

0 1 1 2

1 5 1 6

2 8 6 14

3 10 6 16

4 4 4 8

5 1 1 2

29 19 48

**Table S.2.** Number of viable alleles for each mutation.

**Mutations Viable alleles Average Non-viable Average Total**

**mutations alleles mutations on**

**on viable allele non-viable allele**

I164L 14 3 10 3.4 24

S108N 7 2.6 9 3 16

S108T 12 3 4 3 16

C59R 17 3.6 7 3 24

N51I 14 3.4 10 3 24

A16V 8 3.1 16 3.4 24

**Table S.3.** IC25 and IC2 values for all strains. Values were estimated from regression analysis asymptote and IC50 values.

**Allele log10(IC25) log10(IC2)**

00000 -7.565 -10.555

00001 -6.779 -10.477

00010 -6.386 -10.362

00020 -6.908 -10.096

00021 -6.126 -9.898

00100 -7.372 -9.863

00101 -7.106 -9.769

00110 -4.530 -9.739

00120 -5.602 -9.592

00111 7.230 -9.376

00121 -5.578 -9.116

01000 -6.873 -8.761

01001 -6.602 -8.592

01010 -5.771 -8.568

01011 -4.318 -8.379

01021 -5.389 -8.209

01100 -7.487 -7.724

01101 -6.74 -7.566

01110 -5.219 -7.520

01111 -3.674 -7.310

01121 -4.320 -7.308

10020 -2.726 -7.002

10101 -0.723 -6.664

10120 -4.012 -6.235

10121 -0.046 -5.716

11020 -4.576 -3.713

11100 -4.734 -3.036

11120 -3.245 -4.240

11121 8.356 5.365

**Table S.4.** IC2 as determined by linear regression based on the points bracketing a 2% reduction in growth rate.

**Strain x1, x2 y1, y2 Slope Intercept log10(IC2)**

00111 -10, -7 0.000787, 0.000767 -6.48E-06 0.000722 -7.57E+00

10101 -5, -4 0.000808, 0.000791 -1.66E-05 0.000725 -4.163262

10121 -6, -4 0.000758, 0.000754 -1.87E-06 0.000747 -4.944955

11121 -4, -3 0.000810, 0.000744 -6.57E-05 0.000547 -3.977458

**Table S.5.** All possible pathways from 10^5^ runs on 750 landscapes

**Path Mean SD 95% CI 95% CI Count**

**upper lower**

00000 00010 00110 00111 0.52690 0.02308 0.56323 0.45036 750

00000 00010 01010 01011 01111 00111 0.08765 0.01920 0.15691 0.07630 750

00000 01000 01010 01011 01111 00111 0.06780 0.00853 0.08140 0.05211 750

00000 00020 10020 0.06595 0.01149 0.09785 0.05262 750

00000 00001 01001 01011 01111 00111 0.04636 0.00243 0.04899 0.04130 750

00000 00001 00021 01021 01011 01111 00111 0.03671 0.00677 0.05469 0.02651 750

00000 00100 00110 00111 0.03112 0.00327 0.03332 0.01877 750

00000 00001 00021 01021 01121 11121 0.02118 0.00463 0.03296 0.01524 750

00000 00010 01010 01110 01111 00111 0.02072 0.00304 0.02714 0.01721 750

00000 00001 00021 00121 10121 11121 0.02040 0.00453 0.02961 0.00933 750

00000 01000 01010 01110 01111 00111 0.01620 0.00252 0.02066 0.01123 750

00000 01000 01001 01011 01111 00111 0.01319 0.00126 0.01480 0.01002 750

00000 00001 00021 00121 00111 0.00906 0.00203 0.01317 0.00416 750

00000 00010 01010 01110 00110 00111 0.00787 0.00113 0.01086 0.00586 750

00000 01000 01010 01110 00110 00111 0.00621 0.00120 0.00803 0.00369 750

00000 00001 01001 01021 01011 01111 00111 0.00323 0.00025 0.00367 0.00269 750

00000 00020 00010 00110 00111 0.00234 0.00023 0.00275 0.00187 750

00000 00100 00120 10120 10020 0.00225 0.00034 0.00285 0.00126 750

00000 00001 01001 01021 01121 11121 0.00189 0.00044 0.00260 0.00128 750

00000 00020 00120 10120 10020 0.00164 0.00028 0.00229 0.00120 750

00000 00001 00021 00121 01121 11121 0.00145 0.00034 0.00221 0.00072 750

00000 00100 00120 10120 10121 11121 0.00117 0.00020 0.00151 0.00067 750

00000 00020 00021 01021 01011 01111 00111 0.00102 0.00018 0.00139 0.00071 750

00000 01000 01001 01021 01011 01111 00111 0.00092 0.00013 0.00115 0.00067 750

00000 00001 00021 01021 01121 01111 00111 0.00085 0.00016 0.00121 0.00058 750

00000 00020 00120 10120 10121 11121 0.00084 0.00015 0.00118 0.00059 750

00000 00100 00120 00110 00111 0.00070 0.00012 0.00092 0.00040 750

00000 00020 00021 01021 01121 11121 0.00059 0.00014 0.00088 0.00038 750

00000 00020 00021 00121 10121 11121 0.00057 0.00013 0.00080 0.00028 750

00000 01000 01001 01021 01121 11121 0.00054 0.00015 0.00083 0.00030 750

00000 00020 00120 00110 00111 0.00051 0.00010 0.00074 0.00033 750

00000 00020 00010 01010 01011 01111 00111 0.00039 0.00011 0.00074 0.00025 750

00000 00100 00120 10120 11120 11121 0.00033 0.00008 0.00047 0.00016 750

00000 00020 00021 00121 00111 0.00025 0.00007 0.00039 0.00012 750

00000 00020 00120 10120 11120 11121 0.00024 0.00006 0.00039 0.00013 750

00000 00100 00101 00111 0.00022 0.00006 0.00033 0.00012 750

00000 00100 00101 10101 10121 11121 0.00022 0.00006 0.00032 0.00011 750

00000 00020 00010 01010 01110 01111 00111 0.00009 0.00003 0.00016 0.00003 750

00000 00001 01001 01021 01121 01111 00111 0.00008 0.00003 0.00013 0.00003 749

00000 00001 00021 00121 01121 01111 00111 0.00006 0.00003 0.00012 0.00001 746

00000 00020 00021 00121 01121 11121 0.00004 0.00002 0.00009 0.00001 736

00000 00020 00010 01010 01110 00110 00111 0.00003 0.00002 0.00008 0.00000 730

00000 00100 00120 00121 10121 11121 0.00003 0.00003 0.00009 0.00000 477

00000 00001 00021 00121 00120 10120 10020 0.00003 0.00004 0.00013 0.00000 265

00000 00020 00021 01021 01121 01111 00111 0.00002 0.00002 0.00006 0.00000 665

00000 00020 00120 00121 10121 11121 0.00002 0.00002 0.00007 0.00000 471

00000 01000 01001 01021 01121 01111 00111 0.00002 0.00002 0.00005 0.00000 659

00000 00001 00021 00121 00120 10120 10121 11121 0.00002 0.00002 0.00007 0.00000 259

00000 00100 00120 00121 00111 0.00001 0.00002 0.00005 0.00000 412

00000 00020 00120 00121 00111 0.00001 0.00001 0.00004 0.00000 366

00000 00001 00021 00121 00120 00110 00111 0.00001 0.00001 0.00005 0.00000 247

00000 00100 00101 00121 10121 11121 0.00001 0.00001 0.00003 0.00000 349

00000 00100 00101 00001 01001 01011 01111 00111 0.00000 0.00001 0.00002 0.00000 284

00000 00001 00021 00121 00120 10120 11120 11121 0.00000 0.00001 0.00003 0.00000 186

00000 00100 00101 00001 00021 01021 01011 01111 00111 0.00000 0.00001 0.00002 0.00000 228

00000 00100 00101 01101 01111 00111 0.00000 0.00001 0.00002 0.00000 208

00000 00100 00101 00121 00111 0.00000 0.00001 0.00002 0.00000 167

00000 00100 00101 00001 00021 00121 10121 11121 0.00000 0.00000 0.00001 0.00000 156

00000 00100 00101 00001 00021 01021 01121 11121 0.00000 0.00000 0.00001 0.00000 149

00000 00100 00120 00121 01121 11121 0.00000 0.00000 0.00001 0.00000 118

00000 00020 00021 00121 01121 01111 00111 0.00000 0.00000 0.00001 0.00000 116

00000 00020 00120 00121 01121 11121 0.00000 0.00000 0.00001 0.00000 97

00000 00100 01100 11100 11120 11121 0.00000 0.00001 0.00000 0.00000 8

00000 00100 00101 00001 00021 00121 00111 0.00000 0.00000 0.00001 0.00000 66

00000 00100 00101 01101 01121 11121 0.00000 0.00000 0.00001 0.00000 57

00000 00020 00021 00121 00120 10120 10020 0.00000 0.00000 0.00001 0.00000 46

00000 00100 01100 01110 01111 00111 0.00000 0.00001 0.00000 0.00000 8

00000 00020 00021 00121 00120 10120 10121 11121 0.00000 0.00000 0.00001 0.00000 32

00000 00100 00101 00121 01121 11121 0.00000 0.00000 0.00001 0.00000 30

00000 00100 00101 00001 01001 01021 01011 01111 00111 0.00000 0.00000 0.00001 0.00000 22

00000 00100 01100 01110 00110 00111 0.00000 0.00000 0.00000 0.00000 8

00000 00100 00101 01101 01001 01011 01111 00111 0.00000 0.00000 0.00000 0.00000 18

00000 00100 00101 00001 01001 01021 01121 11121 0.00000 0.00000 0.00000 0.00000 15

00000 00020 00021 00121 00120 00110 00111 0.00000 0.00000 0.00000 0.00000 13

00000 00020 00021 00121 00120 10120 11120 11121 0.00000 0.00000 0.00000 0.00000 11

00000 00100 00101 00001 00021 01021 01121 01111 00111 0.00000 0.00000 0.00000 0.00000 9

00000 00100 00101 00001 00021 00121 01121 11121 0.00000 0.00000 0.00000 0.00000 8

00000 00100 01100 01000 01010 01011 01111 00111 0.00000 0.00000 0.00000 0.00000 5

00000 00020 00120 00121 01121 01111 00111 0.00000 0.00000 0.00000 0.00000 6

00000 00100 00120 00121 01121 01111 00111 0.00000 0.00000 0.00000 0.00000 2

00000 00100 01100 01000 01010 01110 01111 00111 0.00000 0.00000 0.00000 0.00000 2

00000 00100 00101 01101 01001 01021 01121 11121 0.00000 0.00000 0.00000 0.00000 1

00000 00100 00101 00121 00120 10120 10020 0.00000 0.00000 0.00000 0.00000 1

**Table S.6. *Pfdhfr* alleles***

**Allele A16V N51I C59R S108T S108N I164L Viable**

**Wildtype** 00000 0 0 0 0 0 0 *

**1 mutation** 00001 0 0 0 0 0 1 *

00010 0 0 0 0 1 0 *

00020 0 0 0 1 0 0 *

00100 0 0 1 0 0 0 *

01000 0 1 0 0 0 0 *

10000 1 0 0 0 0 0

**2 mutations** 00011 0 0 0 0 1 1

00021 0 0 0 1 0 1 *

00101 0 0 1 0 0 1 *

00110 0 0 1 0 1 0 *

00120 0 0 1 1 0 0 *

01001 0 1 0 0 0 1 *

01010 0 1 0 0 1 0 *

01020 0 1 0 1 0 0

01100 0 1 1 0 0 0 *

10001 1 0 0 0 0 1

10010 1 0 0 0 1 0

10020 1 0 0 1 0 0 *

10100 1 0 1 0 0 0

11000 1 1 0 0 0 0

**3 mutations** 00111 0 0 1 0 1 1 *

00121 0 0 1 1 0 1 *

01011 0 1 0 0 1 1 *

01021 0 1 0 1 0 1 *

01101 0 1 1 0 0 1 *

01110 0 1 1 0 1 0 *

01120 0 1 1 1 0 0

10011 1 0 0 0 1 1

10021 1 0 0 1 0 1

10101 1 0 1 0 0 1 *

10110 1 0 1 0 1 0

10120 1 0 1 1 0 0 *

11001 1 1 0 0 0 1

11010 1 1 0 0 1 0

11020 1 1 0 1 0 0 *

11100 1 1 1 0 0 0 *

**4 mutations** 11120 1 1 1 1 0 0 *

01111 0 1 1 0 1 1 *

01121 0 1 1 1 0 1 *

10111 1 0 1 0 1 1

10121 1 0 1 1 0 1 *

11011 1 1 0 0 1 1

11021 1 1 0 1 0 1

11101 1 1 1 0 0 1

11110 1 1 1 0 1 0

**5 mutations** 11111 1 1 1 0 1 1

11121 1 1 1 1 0 1 *

***** The alleles constructed for these experiments are arranged by number of mutations from the wildtype. Columns include the code for each allele, followed by columns listing the loci where mutations may be present. Wildtype states are indicated by a 0 at that position and mutant states are indicated by a 1 or 2. Thus 00000 is the wildtype and 11111 a quintuple mutant. From left to right the positions correspond to amino acid sites 16, 51, 59, 108 (where 1 is S108N and 2 is S108T), and 164. * indicates alleles that are viable (will grow) in the absence of dTMP supplementation.

**Table S.7. IC50 values of 29 viable alleles and their standard errors and 95% confidence intervals.**

**Allele log10(IC50) SE 95% CI lower 95% CI upper**

00000 -6.741 0.036 -6.770 -6.713

00001 -5.955 0.038 -5.979 -5.930

00010 -5.562 0.023 -5.578 -5.547

00020 -6.084 0.094 -6.152 -6.016

00021 -5.302 0.112 -5.366 -5.239

00100 -6.548 0.049 -6.586 -6.510

00101 -6.282 0.071 -6.336 -6.228

00110 -3.706 0.163 -3.759 -3.654

00120 -4.778 0.114 -4.824 -4.732

00111 > -1.5* - - -

00121 -4.754 0.093 -4.796 -4.711

01000 -6.049 0.022 -6.066 -6.032

01001 -5.778 0.077 -5.827 -5.730

01010 -4.947 0.248 -5.068 -4.826

01011 -3.494 0.080 -3.511 -3.476

01021 -4.565 0.044 -4.588 -4.542

01100 -6.663 0.041 -6.698 -6.628

01101 -5.925 0.028 -5.945 -5.906

01110 -4.395 0.049 -4.423 -4.368

01111 -2.850 0.118 -2.858 -2.841

01121 -3.496 0.928 -3.512 -3.481

10020 -1.902 0.401 -1.943 -1.860

10101 > -1.5* - - -

10120 -3.188 0.077 -3.194 -3.182

10121 > -1.5* - - -

11020 -3.752 0.701 -3.769 -3.736

11100 -3.910 0.015 -3.919 -3.901

11120 -2.421 0.172 -2.426 -2.416

11121 > -1.5* - - -

* indicates the four most resistant strains where IC50 values that are high but indistinguishable given our data

**Figure S.1.** Growth rates of all viable alleles in the absence of drug. The growth rates were determined by measuring cell density at OD600.
